# Bayesian Determination of Parameters for Plasma-Wall Interactions

**DOI:** 10.3390/e21121175

**Published:** 2019-11-29

**Authors:** Roland Preuss, Rodrigo Arredondo, Udo von Toussaint

**Affiliations:** Max-Planck-Institut für Plasmaphysik, 85748 Garching, Germany; Rodrigo.Arredondo@ipp.mpg.de (R.A.); udt@ipp.mpg.de (U.v.T.)

**Keywords:** uncertainty quantification, non-intrusive, spectral expansion, plasma-wall interactions, Bayesian analysis, 02.50.-r, 52.65.-y

## Abstract

Within the Bayesian framework a non-intrusive uncertainty quantification is performed to assess the uncertainty of ion–solid interaction simulations. For this we employ a reduced-order spectral expansion approach which is capable of confining the number of model runs to a feasible size. Moreover, the method facilitates sensitivity examinations regarding to input parameters of the model. It is applied to the ion–solid simulation program SDTrimSP with several uncertain but normally distributed input parameters, i.e., impact angle α, projectile energy $E_{0}$, and surface binding energy $E_{sb}$. Consequently, the otherwise hardly accessible model parameter $E_{sb}$ can be estimated in combination with recently acquired experimental data.

## 1. Introduction

Plasma-wall interactions are of crucial importance in the design of future fusion reactors, since they determine the replacement cycle for the plasma exposed components of the wall. In order to estimate the lifetime of those wall components atomistic simulations are essential. The basic principles of the collisional processes are well understood within the binary collision approximation (BCA) for which the first algorithmic approaches took place already more than 50 years ago [1]. Various sets of parameters are on input to codes simulating ion–solid interactions [2,3,4], as there are composition of the solid, surface roughness, or impact angle of the incident ions. Many of these parameters are uncertain and a proper comparison with experimental data or other models requires the quantification of the uncertainty of the result. Since the employed simulation-based methods of tackling many particles traveling collisionally through matter imply the presence of overall non-linear interactions, an easy access to estimates of the output uncertainties is pointless. Additionally, the computational expense of individual simulations limits the number of simulations runs. Thus the estimation of output uncertainties by Monte-Carlo (MC) methods or grid-based sampling often exceeds the available computational budget, especially if the number of uncertain input parameters is large. Due to the fact that the simulation data come with no estimation of their uncertainties, a sensitivity analysis of a surrogate accomplished by Gaussian processes [5] from the data is costly as well, since it would require repetitive calls of the simulation code to acquire some variance estimates. Therefore, in order to reduce the computational effort we propose for the assessment of uncertainties of plasma-wall interaction simulations a well-known non-intrusive reduced-order model approach (aka polynomial chaos expansion [6]). While the intrusive method, firstly introduced in the context of the stochastic Galerkin finite-element method [7], depends on the formulation and solution of a stochastic version of the original model, which complicates model changes and dresses up the impact of different model terms to the output uncertainty, the non-intrusive method advantageously requires multiple solutions of the original model, only. Consequently, our proposed approach not only reduces the number of function evaluations needed by sampling methods, but provides simultaneously a quantitative measure of which combinations of inputs have the most important impact on the result, i.e., it yields a sensitivity analysis and the associated Sobol coefficients.

## 2. Bayesian Uncertainty Quantification

Based on the Bayesian framework we employ a spectral expansion to quantify the propagation of uncertainty through the model. First introduced by Wiener [8] in the context of Hermite basis functions, it was termed ‘polynomial chaos expansion’ at the time. Nowadays the notion of ‘chaos’ has shifted and the use of the term ‘spectral expansion’ is more appropriate. Once successfully achieved, the spectral representation is capable of quantifying the uncertainty for any point in model space or to serve as a surrogate model.

For a spectral expansion, i.e., the description of a function by an in principle infinite (but in real use finite) number of terms composed of an advantageously orthonormal basis function multiplied by a "spectral" coefficient. Since the spectral coefficients are determined from a discrete set of collocation points in the space of the uncertainty parameters our approach is non-intrusive, but approximate due to the finite number of terms. The emerging integrals in the calculation of the coefficients are evaluated by Gaussian quadrature which identifies the collocation points with those of the quadrature. Moreover, we assume mutually independent normally distributed uncertainty parameters. The probability density function in such a case is Gaussian and the adjunctive set of orthonormal basis functions are Hermite polynomials [9].

To quantify the uncertainty of a result *R* with uncertainty parameters $\vec{\Xi }=\left\{\xi_{1},\xi_{2},\ldots ,\xi_{M}\right\}$, we seek the appropriate function $g(\vec{\Xi })$ such that *R* will have the required distribution of the model response, $R=g(\vec{\Xi })$. It is always possible to find an infinite expansion

(1) $$g\left(\vec{\Xi }\right)=\sum_{k=0}^{\infty }a_{k}\psi_{k}\left(\vec{\Xi }\right)\approx \sum_{k=0}^{P}a_{k}\psi_{k}\left(\vec{\Xi }\right),$$

if the uncertainty parameters $\vec{\Xi }$ have finite variance. The approach is only feasible, if contributions of higher orders become numerically insignificant and the infinite expansion in Equation (1) can be limited to polynomial order *P*. This will be the case if the model preserves the functional nature of the probability density function of the uncertainty parameters, i.e., a normally distributed uncertainty parameter ξ leads to a normal distribution of the model value *R*.

Within the spectral expansion the coefficients $a_{k}$ are defined by

(2) $$a_{k}=\frac{\langle g\left(\vec{\Xi }\right),\psi_{k}\left(\vec{\Xi }\right)\rangle }{\langle \psi_{k}\left(\vec{\Xi }\right),\psi_{k}\left(\vec{\Xi }\right)\rangle },$$

with

(3) $$\langle g\left(\vec{\Xi }\right),\psi_{k}\left(\vec{\Xi }\right)\rangle =\int g\left(\vec{\Xi }\right)\psi_{k}\left(\vec{\Xi }\right)p\left(\vec{\Xi }\right)d\vec{\Xi }=\int g\left(\vec{\Xi }\right)\psi_{k}\left(\vec{\Xi }\right)p\left(\xi_{1}\right)p\left(\xi_{2}\right)\ldots p\left(\xi_{M}\right)d\xi_{1}d\xi_{2}\ldots d\xi_{M}.$$

We assume Gaussian character for the random variable, so the density p(ξ) is distributed according to the normal (probability) distribution

(4) $$p\left(\xi \right)=\frac{1}{\sqrt{2\pi }}\exp\left\{-\frac{\xi^{2}}{2}\right\}.$$

As stated above the adjunctive set of orthonormal basis functions for normally distributed parameters is given by the so-called *probabilist* Hermite functions [9], which read up to fourth order $He_{0}\left(\xi \right)=1$, $He_{1}\left(\xi \right)=\xi$, $He_{2}\left(\xi \right)=\xi^{2}-1$, $He_{3}\left(\xi \right)=\xi^{3}-3\xi$, $He_{4}\left(\xi \right)=\xi^{4}-6\xi^{2}+3$. Because contributions from higher orders become negligible the chosen polynomial order suffices for a good description of the uncertainty. With *M* as the number of the uncertainty parameters and *P* the highest order of the polynomial basis set a total of K=(M+P)!/M!P! coefficients $a_{k}$ have to be determined. For an example with M=2 uncertainty parameters and P=4 one would get following K=15 mixed basis functions $\psi_{k}\left(\vec{\Xi }\right)$:
$\psi_{0}\left(\vec{\Xi }\right)=1$$\psi_{1}\left(\vec{\Xi }\right)=\xi_{1}$$\psi_{3}\left(\vec{\Xi }\right)=\xi_{1}^{2}-1$$\psi_{6}\left(\xi \right)=\xi_{1}^{3}-3\xi_{1}$$\psi_{10}\left(\vec{\Xi }\right)=\xi_{1}^{4}-6\xi_{1}^{2}+3$
$\psi_{2}\left(\vec{\xi }\right)=\xi_{2}$$\psi_{4}\left(\vec{\Xi }\right)=\xi_{1}\xi_{2}$$\psi_{7}\left(\xi \right)=\xi_{1}^{2}\xi_{2}-\xi_{2}$$\psi_{11}\left(\vec{\Xi }\right)=\xi_{1}^{3}\xi_{2}-3\xi_{1}\xi_{2}$

$\psi_{5}\left(\vec{\Xi }\right)=\xi_{2}^{2}-1$$\psi_{8}\left(\xi \right)=\xi_{2}^{2}\xi_{1}-\xi_{1}$$\psi_{12}\left(\vec{\Xi }\right)=\xi_{1}^{2}\xi_{2}^{2}-\xi_{1}^{2}-\xi_{2}^{2}+1$


$\psi_{9}\left(\xi \right)=\xi_{2}^{3}-3\xi_{2}$$\psi_{13}\left(\vec{\Xi }\right)=\xi_{2}^{3}\xi_{1}-3\xi_{2}\xi_{1}$



$\psi_{14}\left(\vec{\Xi }\right)=\xi_{2}^{4}-6\xi_{2}^{2}+3$

The normalization constants in Equation (3) are readily

(5) $$\langle \psi_{k},\psi_{k}\rangle \;=\;\int \psi_{k}\left(\vec{\Xi }\right)\psi_{k}\left(\vec{\Xi }\right)p\left(\vec{\Xi }\right)d\vec{\Xi }\;=\;\max_{k}!.$$

where $\max_{k}$ denotes the highest order in function $\psi_{k}\left(\vec{\Xi }\right)$, e.g., $\max_{k=14}!=4!=24$.

Due to the Gaussian nature of the probability function omnipresent in the integrals above, it is beneficial to use Gauss–Hermite quadrature of order *J* for the evaluation:

(6) $$\langle g\left(\vec{\Xi }\right),\psi \left(\vec{\Xi }\right)\rangle \overset{G.H.}{=}\sum_{j_{1}=1}^{J}\sum_{j_{2}=1}^{J}\ldots \sum_{j_{M}=1}^{J}g\left(\xi_{j_{1}},\xi_{j_{2}},\ldots \xi_{j_{M}}\right)\psi_{k}\left(\xi_{j_{1}},\xi_{j_{2}},\ldots \xi_{j_{M}}\right)w_{j_{1}}w_{j_{2}}\ldots w_{j_{M}},$$

where the weights $w_{j}$ and the abscissas $\xi_{j}$ are provided by e.g., numerical recipes [10]. Eventually, by making use of the orthogonal properties of the probabilist Hermite polynomials the mean of the model result and its variance can be expressed with the spectral coefficients in Equation (3)

(7) $$\langle R\rangle =a_{0}\;,\;\mathrm{var}\left(R\right)\;=\;\langle R^{2}\rangle -{\langle R\rangle }^{2}\;=\;\sum_{k=1}^{K}a_{k}^{2}k!.$$

Sobol coefficients [6] describe the impact of the uncertainty of the input on the result. For first and second order they are defined by

(8) $$S_{i}=\frac{D_{i}}{\mathrm{var}(R)}$$

and

(9) $$S_{ij}=\frac{D_{ij}}{\mathrm{var}(R)},$$

where the evaluation of the respective integrals

(10) $$D_{i}=\int g_{i}^{2}\left(\xi_{i}\right)d\xi_{i},$$

and

(11) $$D_{ij}=\int \int g_{ij}^{2}\left(\xi_{i},\xi_{j}\right)d\xi_{i}d\xi_{j},$$

results in combinations of the coefficients of Equation (3). The first order Sobol coefficients of Equation (8) answer the question which of the input parameters has the largest impact on the uncertainty of the model outcome: the higher the value with respect to the others, the more it is advantageous to reduce the uncertainty of its associated parameter in order reduce the uncertainty of the quantity of interest.

## 3. Ion–Solid Interaction Program SDTrimSP

SDTrimSP [11,12] is a parallelized Monte Carlo code which simulates transport of energetic particles through a target by employing sequentially two-body collision approximation to compute collision cascades in three dimensions. This approximation has been shown to be valid (i.e., the stochastic fluctuations of the collision processes exceed the approximation error) for impact energies larger than about 50 eV [13]. Versions of the SDTrimSP code differ in the description of the target composition, e.g., as one-dimensional (c(x) [12]), two-dimensional (c(x,y) [14]), or three-dimensional (c(x,y,z) [15]). Common to all versions (and key to the high code efficiency) is the assumption of amorphous targets, which circumvents the storage of sample atom coordinates. The simulations were performed with standard settings, i.e., considering a static one-dimensional target (the concentral profile c(x) was kept constant) and the scattering integral was computed using the Gauss–Mehler quadrature scheme with eight pivots. The varied parameters were the projectile energy and the impact angle (with zero degrees corresponding to a perpendicular impact, parallel to the surface normal).

## 4. Results and Discussion

The above program is applied to simulate ion–solid interactions for the case of incident deuterium ions with an energy of $E_{0}=200$ eV at an impact angle of α=45 degrees to a surface consisting of iron with a commonly used surface binding energy of $E_{SB}=4.28$ eV. We assume the parameters to be Gaussian distributed within a σ of about 10%, i.e., $\sigma_{E_{0}}=20$ eV, $\sigma_{E_{SB}}=0.4$ degrees and $\sigma_{\alpha }=4$ eV.

First, in order to have a calibration standard to compare with we employ random sampling of the model response. For each realization of the random variable $\left\{\xi_{1},\ldots ,\xi_{N}\right\}$ there exists a model response $R_{i}=R\left(\xi_{i}\right)$ constituting the sample solution set $\left\{R_{1},\ldots ,R_{N}\right\}$ from which moments can be computed. For this the expected mean is

(12) $$\langle R\rangle =\frac{1}{N}\sum_{i=1}^{N}R\left(\xi_{i}\right)$$

and its variance reads

(13) $$\mathrm{var}\left(R\right)=\langle R^{2}\rangle -{\langle R\rangle }^{2}.$$

**Figure 1:**
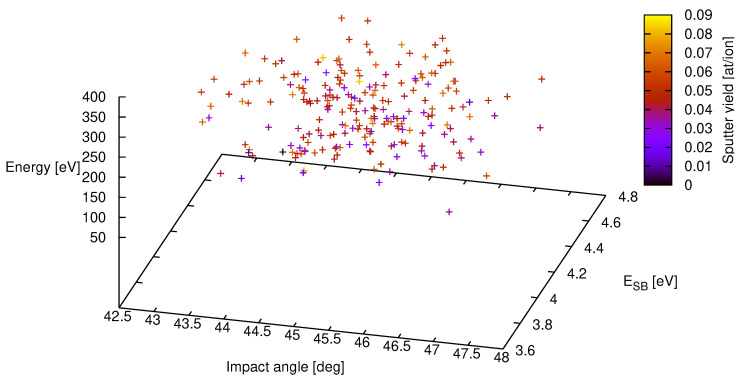
Sputter yield for deuterium on iron from SDTrimSP-simulations with energy, angle and surface binding energy distribution for a subset of 216 out of a total of $20^{3}$ samples. The respective input variables are $E_{0}=200\pm 20$ eV, $E_{SB}=4.28\pm 0.4$ eV and an incident angle *of* 45±4 degrees. The resulting sputter yield is plotted with a color scheme ranging from dark blue at zero up to light yellow at 0.09 sputtered atoms per incoming ion. The mean value of the sputter yield is $Y_{MC}=0.052$ with a standard deviation of $\sigma_{MC}=0.013$.

In Figure 1 the results of a subset of a total of $20^{3}$ = 8000 samples are shown for the above parameter settings. While a depiction of the total set would overcharge the figure it was used to calculated the variances for the yields of the respective parameter settings. The mean value for the sputter yield is $Y_{MC}=0.052$ with a standard deviation of $\sigma_{MC}$ = 0.013. Even more, the full uncertainty distribution may be established with help of a histogram if the sample solution set is sufficiently large (N≳ 1000).

Although this procedure is straightforward and automatically contains the full model answer with all correlations, it has the vital drawback of a comparatively low convergence rate. If the computation time of a single model output is not in the order of seconds or becomes more sophisticated with a higher number of variables (curse of dimension), the mere accumulation of sample point densities to infer the complete distribution is futile.

Much more promising in this respect is the spectral approach of Section 2 which results will be discussed next. Applying the formulas of Section 2 for the case of three uncertainty parameters $\vec{\xi }=\left(\xi_{1},\xi_{2},\xi_{3}\right)$ with

(14) $$\begin{array}{ccc} {\hat{E}}_{0} & = & E_{0}+\xi_{1}\sigma_{E_{0}} \end{array}$$

(15) $$\begin{array}{ccc} {\hat{E}}_{SB} & = & E_{SB}+\xi_{2}\sigma_{E_{SB}} \end{array}$$

(16) $$\begin{array}{ccc} \hat{\alpha } & = & \alpha +\xi_{3}\sigma_{\alpha }, \end{array}$$

the summation of the terms in Equation (6) runs over three indices $l_{1}$, $l_{2}$, and $l_{3}$. It is good numerical praxis to employ the Gaussian quadrature with one order higher [10] than the highest polynomial order of the spectral expansion, which requires an upper boundary of P+1=5 for the used fourth order polynomials.

For numerical accuracy of the Gaussian quadrature it is expedient to be one order higher than the polynomial order of the spectral expansion.

**Figure 2:**
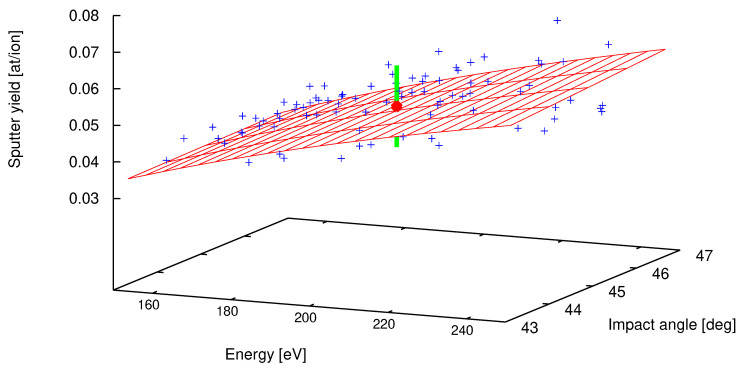
The iron erosion by deuterium sputtering as function of impact angle and energy. The surrogate model is given by the mesh (in red). The polynomial chaos expansion was computed around $E_{0}=200$ eV, $E_{SB}=4.28$ eV, and impact angle of 45 degrees. The red bullet indicates the mean sputter yield of 0.050 with its uncertainty of 0.011 as the light green line. For illustration purposes the input parameters $E_{0}$ and α, while the surface binding energy $E_{SB}$ was set to 4.28 eV. In addition, the blue plus signs show the scatter data from the sampling approach already shown in Figure 1.

This results in a total of 216 terms (three nested summations, each running from $l_{i}$ = 0–5 with *i* = 1,2,3) over the collocation points composed of six Gaussian quadrature abscissas assigned to $\xi_{l_{i}}$ and six weights $w_{l_{i}}$. The value for the function $g(\xi_{l_{1}},\xi_{l_{2}},\xi_{l_{3}})$ is obtained from a SDTrimSP run, which takes roughly three minutes on a modern CPU. However, the computations can be accelerated tremendously because the simulations can be run in parallel. Once calculated, the 35 coefficients of Equation (6) establish a fast surrogate model, which is simply the evaluation of a polynomial. This is shown in Figure 2 as the red mesh. The respective sputter yield, for which the uncertainty quantification was performed, is depicted in the center as the red sphere with $Y_{UQ}=0.050$ at/ion and its standard deviation of $\sigma_{UQ}=0.011$ as the green perpendicular line. The comparison with the result of the sampling approach above ($Y_{MC}=0.052\pm 0.013$) shows excellent agreement.

Without the need to do any further simulations, various quantities may be inferred from the coefficients, e.g., the variance as in Equation (3), or the Sobol coefficients, which allows the investigation of the sensitivity of the result on the uncertainty of the input variables. For the above variables $E_{0}$, α and $E_{SB}$ we get the Sobol coefficients (only first order is numerically significant) shown in Table 1. Regardless of the setting of the surface binding energy $E_{SB}$, the Sobol coefficients indicate that the improvement of the knowledge of $E_{SB}$ is most rewarding if one wants to reduce the uncertainty of the sputter yield.

Following this trail, we performed a series of experimental measurements of the sputter yield for different impact angles with α = 0, 45, 60 and 75 degrees at $E_{0}$ = 2 keV deuteron. The experimental details of the sample preparation, the high-current source SIESTA and of the measurements performed are given in [16]. These angle-resolved data were augmented with energy dependent sputter yield measurements from a recent study [17]. Then we applied the uncertainty quantification method discussed above in order to provide quantitative estimates of the sputter yields at a variety of settings for the surface binding energy $E_{SB}$. It turned out that the most probable value for the surface binding energy is $E_{SB}^{\mathrm{new}}=4.8\pm 0.4$ eV, one and a half standard deviations larger than the value commonly used up to now [4], i.e., $E_{SB}^{\mathrm{old}}=4.2\pm 0.4$ eV.

**Figure 3:**
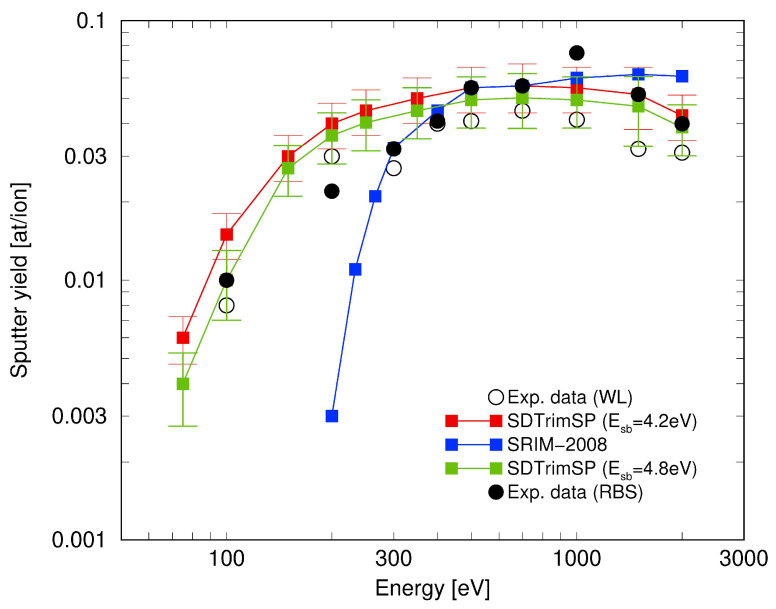
Comparison of the sputter yield as a function of projectile energy for the previously set surface binding energy $E_{SB}=4.2\pm 0.4$ eV (red squares) and the newly acquired setting of energy $E_{SB}=4.8\pm 0.4$ eV (green squares) with data from experiments done in a Rutherford backscattering setup (RBS, filled circles) and a weight loss setup (WL, open circles) [17]. A further model, SRIM (blue squares), can almost certainly be ruled out. All lines shown are guides for the eye.

With the revised setting of $E_{SB}$ we compared (see Figure 3) simulations of the sputter yield for different incident energies of deuterium with results from Rutherford backscattering (RBS) and weight loss (WL) experiments and got an improved agreement (except for $E_{0}$ = 1 keV). With these results the Bayes factor rules out another competitor to SDTrimSP (i.e., Monte Carlo decision for the occurrences of collisions of incident ions with atoms in the target) being the SRIM model, which employs a quantum mechanical treatment of ion-atom collisions and seems not to comprise all important effects present.

## 5. Summary and Conclusions

The non-intrusive polynomial chaos expansion for quantifying the propagation of uncertainty through the model has been proven to be a valuable tool in describing the reliability of a model outcome. The experience with the employed algorithm revealed that the spectral expansion with moderate settings of employing only up to 4th order polynomials and six Gaussian quadrature abscissa, which requires less than 1000 simulation runs, is well suited for the determination of a medium number of uncertain parameters. We applied the method to SDTrimSP simulations in determining the sputtering yield and its standard deviation for the example of incident deuterium ions on an iron target. Residing on both quantities we could rule out the existing parameter setting for the surface binding energy and assigned a new much more accurate one. With this newly set input parameter it was possible to get a better agreement with available experimental data and eventually put us in the position to rule out a competitive physical model.

## Figures and Tables

**Table 1:** The table shows the Sobol coefficients of the three parameters $E_{0}$, $E_{SB}$ and α at a projectile energy of $E_{0}$ = 200 eV and an impact angle of α = 45 degrees for different settings of the surface binding energy $E_{SB}$.

$E_{\mathrm{SB}}$	4.08	4.28	4.48	4.68	4.88	5.08
$S_{E_{0}}$	0.005	0.005	0.006	0.006	0.005	0.007
$S_{E_{SB}}$	0.752	0.734	0.713	0.688	0.666	0.637
$S_{\alpha }$	0.238	0.256	0.277	0.302	0.325	0.352
